# Immunohistochemical Expression of Ki67 and p53 in Wilms Tumor and Its Relationship with Tumor Histology and Stage at Presentation

**DOI:** 10.1155/2016/6123951

**Published:** 2016-01-20

**Authors:** O. H. Radhika Krishna, Geetha Kayla, Mohammed Abdul Aleem, Ramani Malleboyina, Ramesh Reddy Kota

**Affiliations:** ^1^Department of Pathology, Niloufer Hospital for Women and Children, Red Hills, Hyderabad, Telangana 500004, India; ^2^Department of Pediatric Surgery, Niloufer Hospital for Women and Children, Red Hills, Hyderabad, Telangana 500004, India

## Abstract

*Aim*. Evaluate tumor proliferation marker (Ki67) and p53 tumor suppressor marker in Wilms tumor and correlate with histology, anaplasia, and staging.* Design*. Prospective, hospital based study conducted at a tertiary pediatric referral centre in south India.* Setting*. Wilms tumor is the most common childhood renal malignancy worldwide. Anaplasia on histology is associated with treatment resistance but not with aggressiveness clinical presentation. Chemotherapy for Wilms tumor is based on histology and staging. Most patients respond to current chemotherapy protocol. However, a small fraction relapses or metastasizes. Affordable prognostic markers are needed for histopathological evaluation of this tumor.* Subjects*. Cases of histologically confirmed Wilms tumor over five years. Cases after chemotherapy were excluded as the immunostaining was inconsistent in necrotic areas.* Methods*. The clinical and radiological findings of 31 cases of Wilms tumor were documented at a tertiary pediatric referral hospital over five years. In addition to Hematoxylin and Eosin staining, Ki67 proliferation index and p53 expression were correlated with tumor histology and staging.* Results*. Age incidence was 3–8 years with female preponderance. Significant correlation was noted between Ki67 proliferation index and tumor staging. p53 expression was not useful in stratification of Wilms tumor.* Conclusion*. Ki67 was cost-effective immunohistochemical marker for prognostication of pediatric Wilms tumor.

## 1. Introduction

Wilms tumor, the most common renal tumor of children, is known to be associated with chromosomal abnormalities [1]. It arises from the metanephric blastemal cells and recapitulates embryogenesis.

Chemotherapy protocol for Wilms tumor is based on tumor histology and tumor staging. Favourable or unfavourable histology has an impact on tumor prognosis. Stage I or low risk nephroblastoma receives no postoperative treatment while high risk tumors with diffuse anaplasia and blastemal types are treated with aggressive chemotherapy. Most patients respond to current chemotherapy protocol. However a small fraction relapses or metastasizes. There is therefore a need to identify ideal cost-effective prognostic markers for this very common tumor.

A large number of prognostic markers in nephroblastoma have been reviewed [2, 3]. Our study aims to evaluate the efficacy of two cost-effective immunohistochemical markers, tumor proliferation marker (Ki67) and tumor suppressor immunohistochemical marker p53. Their expression was correlated with tumor histology and staging.

## 2. Material and Methods

Case records of 31 cases of Wilms tumor were collected over a period of five years at a tertiary pediatric referral hospital. The clinical history and radiological investigations were documented in all the cases. Cases which had received prior chemotherapy were excluded from the study, since they contained large areas of necrosis and haemorrhage and were not amenable for immunohistochemistry.

All the specimens were received in formalin. A detailed macroscopic examination of the tumor was performed and adrenal glands, lymph nodes, renal vessels, renal sinus, capsule, and pelvic ureter wherever removed along with the kidney tumor were examined and sections were taken to make a pathological staging according to NWTS-5 staging [4].

Hematoxylin and Eosin stained sections were used for tumor diagnosis, histological and tumor staging. Further 3 *μ*m sections were taken on positively charged slides for IHC with Ki67 and p53.

IHC was performed for Ki67 and p53 using standard IHC protocol. Ki67 antigen is a cell proliferation related nonhistone nuclear protein that can be labelled with monoclonal antibody MIB-1. The antigen is expressed in the active phases of the cell cycle. The Ki67 proliferation index is an excellent marker to recognize rapidly proliferating cells. A proliferative index was estimated and defined as percentage of positive Ki67 cells over 5 high power fields. It was semiquantitatively graded as low, moderate, or high and correlated with histological staging.

p53 is a 53 kDa nuclear protein product of tumor suppressor gene p53, located on short arm of chromosome 17. Mutations of p53 are common in human malignancies leading to tumor growth; detectable immunostaining for p53 differentiates malignant cells. p53 was semiquantitatively assessed as mild, moderate, or marked.

## 3. Results

A total of 31 cases of Wilms tumor were studied. Peak age of incidence was 3–8 years. There was significant female preponderance with a male to female ratio of 1 : 1.8.

Based on the NWTS-5 classification, of the 31 cases, 16 cases were in stage I, 11 cases were in stage II, two cases were in stage III, and one case was in stage IV and there was no case in stage V (Table 1).

**Figure 1:**
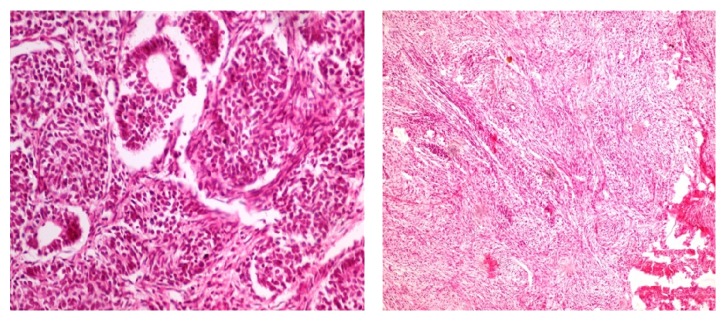
Photomicrograph showing a triphasic Wilms tumor and one with predominant mesenchymal component.

29 cases were triphasic Wilms tumor and three were biphasic. 22 cases showed favourable histology and remaining cases showed unfavourable histology (Figure 1).

**Figure 2:**
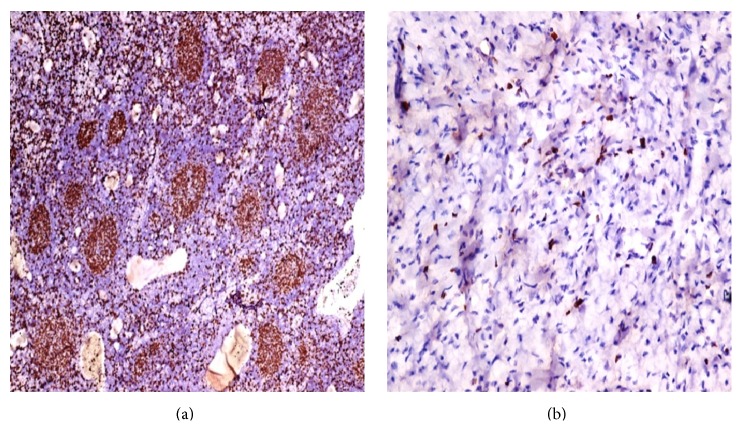
Blastemal component (a) shows higher Ki67 proliferation index as compared to mesenchymal component (b).

Ki67 proliferation index was calculated and correlated with histology and with tumor staging. It ranged from 38 in stage I, 50 in stage II, 69 in stage III, and 79 in stage IV (Table 2). In all the stages the blastemal component showed higher Ki67 proliferation index as compared to the epithelial component (Figure 2).

**Figure 3:**
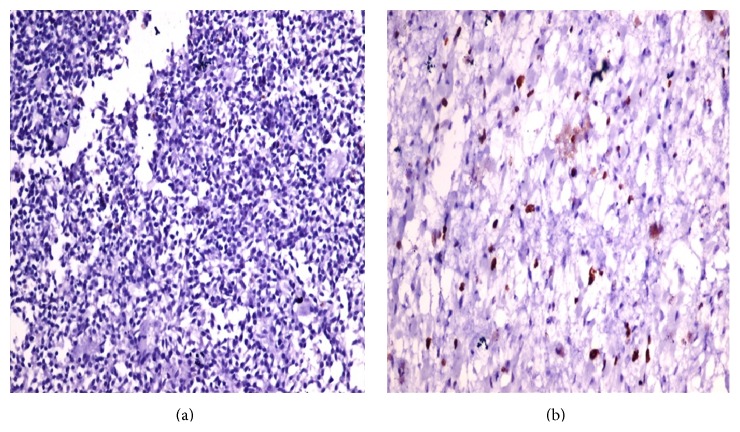
Photomicrograph of stage I Wilms tumor (a) showing nil p53 positivity and a case in stage III (b) showing moderate positivity.

The expression of p53 was expressed as mild, moderate, or marked. No difference was noted in the degree of expression in epithelial and blastemal components (Figure 3). Two cases in stage III and one case in stage IV showed moderate positivity while stages I and II cases were almost negative (Table 3).

## 4. Discussion

31 cases of Wilms tumor were studied over a period of 5 years. Median age incidence was 3 years, slightly lesser than worldwide incidence. An Indian study by Mishra et al. found a median age incidence of 2.5 years [5]. Other Asian studies showed similar age incidence [6]. Sex incidence showed female preponderance, while other Asian studies showed a male preponderance [6]. A NWTS study done over a large population group however showed a female preponderance [7]. This might be a reflection of our social system where more female children are brought to the government hospitals. Tumors with favorable histology may present with aggressive behavior and therefore a need for prognostic evaluation of Wilms tumor.

Most of the cases, 16 out of 31, presented in stage I and 12 in stage II, 2 in stage III, and 1 in stage IV. Staging corresponds to other studies [6].

Dominant histological pattern was triphasic. Unfavourable histology was seen in 15 percent of cases as against western studies which showed only 5 percent [8]. This may be due to delayed presentation of the cases. Indicators of poor prognosis are anaplasia and tumor staging, and anaplasia on histology is subjective.

An attempt was made to characterize the cases through IHC in resource constrained government health centre. Two easily available markers Ki67 and p53 were evaluated. Ki67, tumor proliferation marker, is an important prognostic factor in a variety of cancers [9].

Ki67 is a nuclear antigen associated with cell proliferation, is present throughout the cell cycle, and is absent in resting cells. High Ki67 proliferation index (PI) is associated with a more aggressive clinical behaviour and is found to be a significant determinant of distant metastasis and tumor related deaths [9]. The study found higher Ki67 proliferative index in higher stages of Wilms tumor. Higher stage being associated with a higher index is well known as proven by Juric, Khine, Ghanem, and Juszkiewicz [8, 10–12]. This fact is once again reiterated. However, unlike the previous studies, proliferative index of the blastemal component was significantly high in our study; this can be explained based on the fact that the poorly differentiated blastema expresses higher PI than the well differentiated mesenchymal component. Juszkiewicz found similar results [12]. Das et al. in their study found higher index in the mesenchymal component. PI was higher in the anaplastic areas of the tumor. These findings show that greater proliferation is seen in advancing stage. Juric et al. in their study involving 48 cases of Wilms tumor supported the conclusion that Ki67 is a relevant marker for assessing the proliferative activity and tumor cell dynamics of nephroblastoma and that it may not be a good clinical prognostic marker [8]. Ghanem et al. observed higher PI indicative of clinical progression and poor survival [2]. The role of Ki67 as a proliferative marker in Wilms tumor stands reinforced again.

The role of p53 in the pathogenesis and progression of Wilms tumors is only partly understood [13]. Significance of p53 expression in Wilms tumor is conflicting. Wilms tumor has been associated with chromosomal abnormalities at the 11p13, 11p15, and 16q regions. A study into the possibility of mutations occurring within p53, the ubiquitous adult tumor suppressor gene, was made [14]. Several studies including one by Malkin et al. found the p53 mutations in Wilms tumor [15]. Cheah et al. in their Asian study mentioned that the immunohistochemical expression of p53 protein in Wilms tumor was presumably a result of mutation in the p53 tumor suppressor gene and correlates with histological classification [14]. The accumulation of p53 in these tumors may not only be due to mutations but also involve stabilization of normal p53 with other proteins, histological categorization being one of the useful features in the prognostic assessment of Wilms tumor [13].

However we found low to nil expression of p53 immunostaining in lower stages and moderate expression in higher stages. Similar findings were noted by Das and Zabolinezad in their study [1, 6]. This study does not find immunohistochemical expression of p53 utility in stratification of Wilms tumor. Skotnicka-Klonowicz et al. in their study found significant correlation between p53 expression and tumor staging [16]. All the relevant clinical and histopathological findings of our study including immunostaining are comparable with a similar north Indian study by Das et al. [6].

Therefore, these biological predictors may potentially provide the clinical oncologist with a biological rationale in identifying patients at high risk of tumor recurrence and to guide the adjuvant chemotherapy and/or radiotherapy [17].

## Figures and Tables

**Table 1:** Staging of the tumor.

Stage of the tumor	Number of cases (*n* = 31)
Stage I	16 (51.6%)
Stage II	12 (39%)
Stage III	2 (6%)
Stage IV	1 (3%)
Stage V	0

**Table 2:** Ki67 proliferation index versus NWTS staging.

Stage of tumor	Epithelial	Mean	Blastema	Mean	Average
I (*n* = 16)	14–46	30	24–68	46	38
II (*n* = 12)	32–40	36	66–82	74	55
III (*n* = 2)	60		78		69
IV (*n* = 1)	76		82		79
V	0				

**Table 3:** p53 expression in Wilms tumor.

Stage of the tumor	Number of cases (*n* = 31)
Stage I	Low
Stage II	Low
Stage III	Moderate
Stage IV	Moderate

## Key Messages

Patients with favourable histology show aggressive clinical course. A need for objective assessment of tumor grade by prognostic markers is needed for better judgement of patient prognosis. Ki67 labelling index was found useful in prognostication of Wilms tumor by our study. However we did not find p53 immunostaining of any utility in Wilms tumor.

We advocate the routine use of Ki67 proliferative index in assessment of Wilms tumor histology.
